# Neutrophil extracellular traps predict poor survival in cancer: a systematic review and meta-analysis of studies on tissue and circulating biomarkers

**DOI:** 10.3389/fimmu.2025.1676854

**Published:** 2025-10-03

**Authors:** Seungwoo Lee, Eun Young Kim, Woohyun Park, Young Sub Lee, Kwangil Yim

**Affiliations:** ^1^ Department of Data Science, The Catholic University of Korea, Gyeonggi-do, Republic of Korea; ^2^ Department of Surgery, Uijeongbu St. Mary Hospital, College of Medicine, The Catholic University of Korea, Seoul, Republic of Korea; ^3^ Department of Hospital Pathology, Eunpyeong St. Mary Hospital, College of Medicine, The Catholic University of Korea, Seoul, Republic of Korea; ^4^ Department of Hospital Pathology, Uijeongbu St. Mary Hospital, College of Medicine, The Catholic University of Korea, Seoul, Republic of Korea

**Keywords:** neutrophil extracellular traps, neoplasms, prognosis, systematic review as topic, meta-analysis as topic

## Abstract

**Background:**

Neutrophil extracellular traps (NETs) are fibrous, web like chromatin structures released by activated neutrophils that entrap and immobilize pathogens through histones, granule derived proteolytic enzymes, and myeloperoxidase (MPO) dependent mechanisms. Beyond host defense, NETs have been implicated in tumor progression; yet anticancer activity also has been reported, and findings vary across specimen types (tumor tissue versus blood) and detection methods, antibody panels, leaving their role in oncogenesis uncertain. We performed a systematic review and meta-analysis to define the prognostic significance of NETs in cancer, stratified by specimen type, detection technique, and antibody panels.

**Methods:**

Following PRISMA guidelines, we searched PubMed, EMBASE, and the Cochrane Library for studies published through August 10, 2023, that reported quantitative NET measurements linked to oncologic outcomes.

**Results:**

Fifteen studies (5,202 patients; publication years 2016–2023) reporting hazard ratios (HRs) for overall survival (OS) and disease free survival (DFS) relative to NET levels met inclusion criteria. Six studies evaluated tumor derived NETs in tissue and nine assessed circulating NETs in blood. Among tissue studies, two used immunohistochemistry for citrullinated histone H3 (H3Cit) alone, and four applied multiplex immunofluorescence for MPO/H3Cit or neutrophil elastase (NE)/H3Cit. Among blood studies, enzyme linked immunosorbent assays targeting MPO/DNA predominated, followed by H3Cit assays. Higher NET levels were significantly associated with worse OS (HR 1.80; 95% CI 1.35–2.41) and DFS (HR 2.26; 95% CI 1.82–2.82), irrespective of tissue or blood based measurement. Prognostic associations were robust for MPO/DNA, H3Cit, and NE, but not for cell free DNA.

**Conclusion:**

Elevated NET levels predict poorer outcomes in patients with cancer independent of specimen source and most analytic modalities (except cell free DNA), supporting NETs as a promising biomarker for risk stratification and precision oncologic decision making.

**Systematic review registration:**

https://www.crd.york.ac.uk/PROSPERO/view/CRD42025596821.

## Introduction

Neutrophils are the first responders of the innate immune system, and they play a pivotal role not only in defending the host against invading pathogens (1, 2) but also in modulating the tumor microenvironment and influencing cancer progression (3). In addition to their conventional antimicrobial functions, recent attention has been focused on neutrophil extracellular traps (NETs) and fibrous web-like chromatin structures released by activated neutrophils (4–7). NETs contribute to host defense by entrapping and immobilizing pathogens through a process that relies on histones, granule-derived proteolytic enzymes, and myeloperoxidase (MPO) (6, 8).

Emerging evidence has highlighted the pro-tumorigenic role of NETs in various malignancies (8–10). This role is primarily attributed to their involvement in cellular injury and tissue regeneration, which in turn trigger excessive inflammatory responses (8–10). NETs have been reported to facilitate tumor cell proliferation (11), metastatic dissemination (12–14), immune evasion (15), and cancer-associated thrombosis (16–18).

Nevertheless, NETs have also been reported to exert antitumor effects in certain contexts, and their functional outcomes appear to vary according to tumor type and microenvironmental conditions (19). Moreover, studies investigating the prognostic effect of NETs in cancer have used different sample sources, including blood and tumor tissues. A wide range of detection methods, such as immunohistochemistry (IHC) (20, 21), immunofluorescence (IF) (22–25), and enzyme-linked immunosorbent assay (ELISA) (11, 26–33) have been employed using diverse antibodies, including citrullinated histone H3 (H3Cit) (20, 21, 26–29), MPO/H3Cit (22–24), neutrophil elastase (NE)/H3Cit (25), MPO/DNA (11, 27, 28, 30, 31), NE (27, 29, 32), and cell-free DNA (cfDNA) (26, 27). This methodological variability significantly contributes to heterogeneity in results, complicating the interpretation and comparison of results across studies.

We aimed to address the current literature gaps by systematically analyzing the prognostic relevance of NETs in cancer. We specifically evaluated the heterogeneous findings of NET-related studies by stratifying our analyses based on sample source (tissue vs. blood), detection methodologies, and antibody selection. Through this comprehensive meta-analysis, we aimed to deepen our understanding of the role of NETs in cancer progression and contribute to future clinical applications, including the development of NET-targeted therapeutic strategies.

## Methods

### Search strategy

This meta-analysis was prospectively submitted to PROSPERO (CRD42025596821) and was approved by the Institutional Review Board of the Catholic University of Korea, College of Medicine (UC22ZASI0033). A comprehensive literature search of relevant English-language articles published up to August 10, 2023, was conducted across three major electronic databases (PubMed, EMBASE, and the Cochrane Library) using the search strategy outlined in **Supplementary Table S1**. Additionally, a manual search was performed by screening the reference list of a key article (10). Potentially relevant titles were cross-checked with records from the database search, and any unmatched studies underwent full-text review in accordance with the predefined inclusion and exclusion criteria. EndNote X20 (Build 10136; Thomson Reuters, New York, NY, USA) was used to manage the retrieved studies.

### Inclusion and exclusion criteria

This meta-analysis applied the following inclusion criteria: 1) studies on the relationship between NETs and prognosis of patients with cancer was assessed; 2) NETs identified with accurate examination; 3) studies that provided sufficient information on hazard ratios (HRs) of patient survival; 4) studies that demonstrated an association between NETs and clinicopathological features; and 5) articles written in English language. The following exclusion criteria were applied: 1) duplicate studies, reviews, case reports, letters, and conference proceedings; 2) studies that did not show an association between NETs and survival or clinicopathological parameters; 3) studies related to cancer cell lines and animal models; and 4) studies with insufficient data on HRs and 95% confidence intervals (CIs) that could be extracted or calculated.

### Data extraction and assessment of study quality

Data extraction was performed by five independent reviewers (S. L., E.Y.K., W.P., Y.S.L., and K.Y.). In cases of disagreement, consensus was reached among them. The following data were extracted from all included studies: author, year, ethnicity, number of patients, antibody, detection method, organ, sample type, pathological stage, and survival outcomes such as overall survival (OS) or disease-free survival (DFS). Risk of bias was assessed, and studies that met the inclusion criteria were selected using the Quality in Prognostic Studies tool. In studies without HRs, we used data on the Kaplan–Meier curve to calculate the HR using the method described by Parmar et al. (34).

### Statistical analysis

Statistical analysis was conducted using Review Manager Software (version 5.4.1; Cochrane Collaboration, Copenhagen, Denmark). Pooled HRs with 95% CIs were used to assess the association between NETs and OS. HRs >1 indicated poor survival, whereas those <1 indicated better survival. The association between NETs and other clinicopathological parameters was analyzed using the Mantel–Haenszel pooled odds ratio (OR) with 95% CIs and combined effective value. An I^2^ value of <50% indicated no heterogeneity among the studies. A subgroup analysis was conducted to explore potential sources of heterogeneity. The Preferred Reporting Items for Systematic Reviews and Meta-Analysis flow diagram and forest plots were generated using Review Manager software.

## Results

### Eligible studies

**Figure 1:**
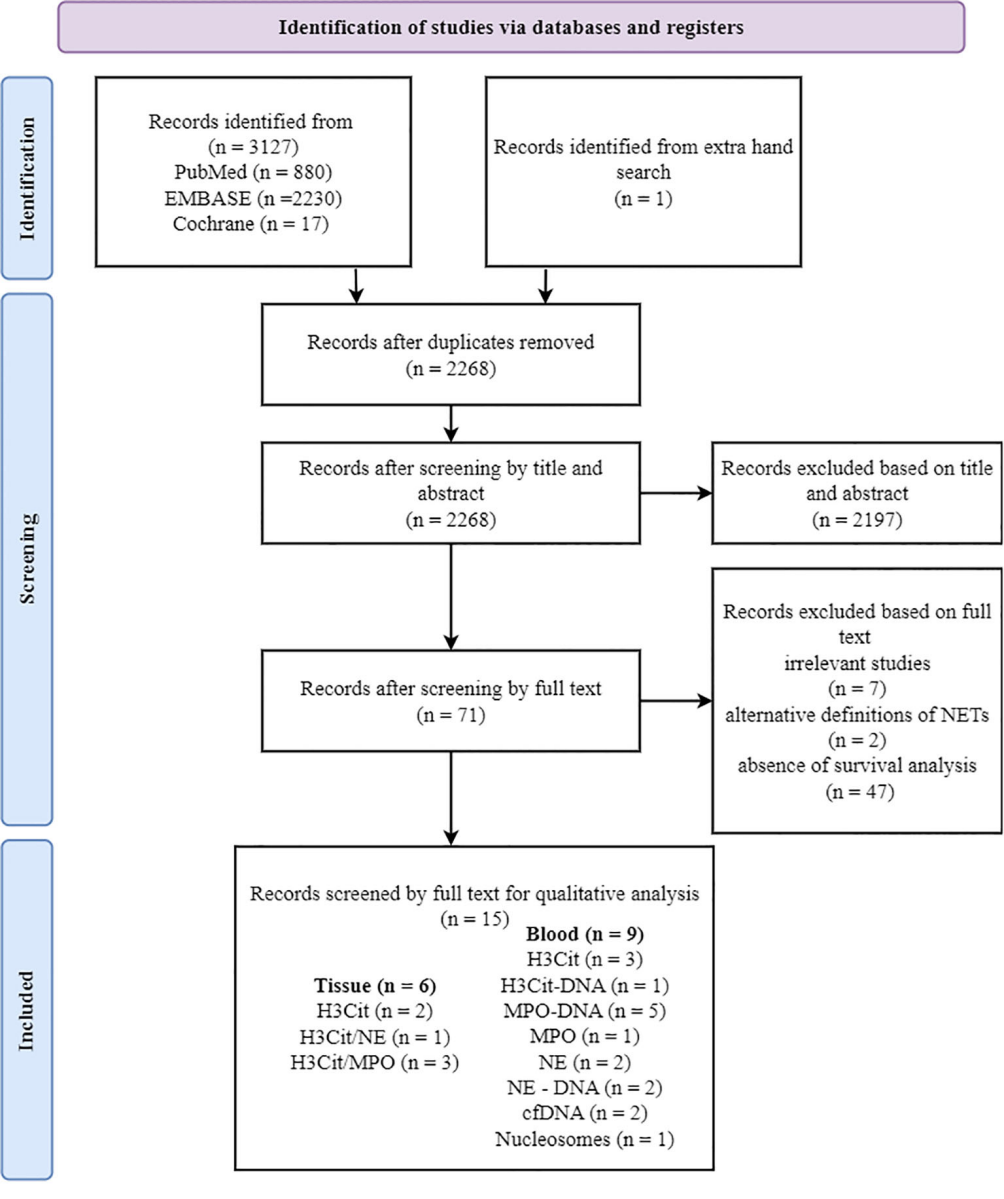
PRISMA flow diagram showing the study selection process. Of 15 studies included in the qualitative analysis, some utilized co-staining approaches (e.g., H3Cit/NE and H3Cit/MPO). The total number of markers exceeds the number of studies, as some studies analyzed multiple antibodies. PRISMA, Preferred Reporting Items for Systematic Reviews and Meta-Analyses; H3Cit, citrullinated histone H3; MPO, myeloperoxidase; NE, neutrophil elastase.

An initial literature search included 3,130 articles from PubMed, EMBASE, and the Cochrane Library (**Figure 1**). From the reference list, several articles were initially considered potentially relevant, but nearly all had already been captured through our database searches, supporting the robustness of our strategy. One additional article mentioned “angiogenesis,” yet full-text assessment revealed that it was study on cancer cell lines and therefore did not meet our inclusion criteria. After removing 862 duplicate articles, the remaining 2,268 articles were screened based on the reference type criteria. The study by Wang et al. (35) was excluded from the systematic review owing to discrepancies among in figure legends, corresponding graphical data, and main text descriptions. Considering available data on prognosis, clinicopathological parameters, evaluation methods, and their association with NETs, 15 articles met the inclusion criteria (**Figure 1**). Most of the studies showed a low risk of bias (**Supplementary Figure S1**).

### Study characteristics

Fifteen studies were selected for the final analysis that investigated the relationship between NETs and survival rates. These studies were conducted in seven different countries and published between 2016 and 2023 (**Table 1**; **Supplementary Table S2**). Among them, studies analyzing progression-free survival (PFS) (22, 29) and cancer-specific survival (CSS) (22) were limited to two and one, respectively, making further analysis challenging (**Table 1**). The total number of patients included was 5,202, with individual study sizes ranging between 27–954 (**Table 1**; **Supplementary Table S2**). The patients were divided into groups with high and low NET levels for comparative analysis.

**Table 1:** Main characteristics of all NET studies included in the meta-analysis.

Sample type	Authors year	Patients (n)	Ethnicity	Organ	Detection method	Antibody	Cut-off	Survival type
Tissue	Shinde-Jadhav et al., 2021 (25)	104	Caucasian	Bladder	Multiplex IF	NE/H3Cit	Presence	OS
	Xu et al., 2021 (21)	135	Asian	Hepatobiliary pancreas (Pancreas)	IHC	H3Cit	Presence	OS
	Yan et al., 2021 (23)	126	Asian	Uterine cervix	Multiplex IF	MPO/H3Cit	Quartile 3 (≥75%)	DFS
	Chen et al., 2022 (22)	205	Asian	Hepatobiliary pancreas (Pancreas)	Multiplex IF	MPO/H3Cit	Presence	PFS, CSS
	Jiang et al., 2022 (20)	80	Asian	Hepatobiliary pancreas (Liver)	IHC	H3Cit	>8 cells/HPF (x400)	DFS, OS
	Zhong et al., 2023 (24)	174(training) 66(validation)	Asian	Gastrointestinal (Rectum)	Multiplex IF	MPO/H3Cit	Median (≥50%)	DFS
Blood	Tohme et al., 2016 (31)	35	Caucasian	Gastrointestinal (Rectum)	ELISA	MPO/DNA	Median (≥50%)	DFS
	Thålin et al., 2018 (27)	60	Caucasian	Various malignancies	ELISA	cfDNA, H3Cit, MPO, MPO/DNA, NE	Quartile 3 (≥75%)^a^	OS
	Grilz et al., 2019 (26)	957	Caucasian	Various malignancies	ELISA	cfDNA, H3Cit, Nucleosomes	Quartile 3 (≥75%)^b^	OS
	Yazdani et al., 2019 (11)	27	Caucasian	Gastrointestinal (Rectum)	ELISA	MPO/DNA	Median (≥50%)	OS
	Zhang et al., 2020 (29)	53	Asian	Gastrointestinal (Stomach)	ELISA	H3Cit, NE	Median (≥50%)	PFS
	Rosell et al., 2021 (32)	106	Caucasian	Various malignancies	ELISA	H3Cit/DNA, NE	Not available	OS
	Li et al., 2023 (30)	80	Asian	Gastrointestinal (Stomach)	ELISA	MPO/DNA	Median (≥50%)	DFS, OS
	Martinez–Cannon et al., 2023 (33)	40	Caucasian	Breast	ELISA	NE/DNA	>0.6705 optical density	DFS^c^
	Okamoto et al., 2023 (28)	133 (H3Cit) 67 (MPO/DNA)	Asian	Gastrointestinal (Stomach)	ELISA	H3Cit, MPO/DNA	Median (≥50%)	DFS, OS

OS, overall survival; DFS, disease-free survival; PFS, progression free survival; CSS, cancer specific survival; IHC, immunohistochemistry; IF, immunofluorescence; ELISA, enzyme-linked immunosorbent assay; cfDNA, cell-free DNA; NETs, neutrophil extracellular traps; H3Cit, citrullinated histone H3; MPO, myeloperoxidase; NE, neutrophil elastase; ^a^cfDNA: ≥597.5ng/ml, H3Cit: ≥29.8ng/ml, MPO: ≥213.4ng/ml, MPO/DNA: not shown, NE: ≥110 ng/ml; ^b^cfDNA: >442.6ng/ml, H3Cit: >87.8ng/ml, Nucleosomes: >3.0 multiple-of-the-median, MPO/DNA: not shown, NE: ≥110 ng/ml; ^c^The hazard ratio was calculated them from Kaplan–Meier curve data using the method described by Parmar et al.

### High NETs levels and prognosis in patients with solid cancer

**Figure 2:**
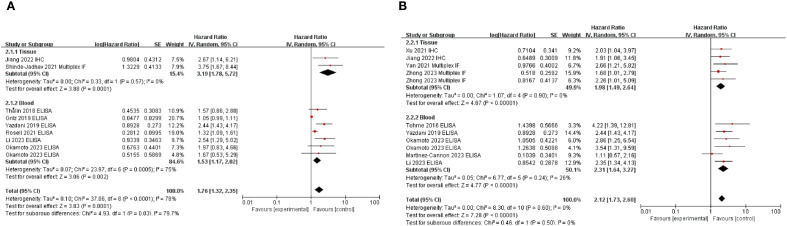
Subgroup analysis of neutrophil extracellular traps according to sample source: overall survival **(A)** and disease-free survival **(B)** in patients with cancer. Zhong et al. provided multivariate analysis results for both training and validation cohorts, whereas Okamoto et al. analyzed patient groups using H3Cit and MPO-DNA antibodies. Additionally, data from studies by Yazdani et al., Okamoto et al., Li et al., and Jiang et al., who reported OS and DFS outcomes, were included in the analysis. High NET levels of neutrophil extracellular traps were associated with poor survival outcomes in both OS and DFS. OS, overall survival; DFS, disease-free survival; NETs, neutrophil extracellular traps.

We evaluated the correlation between NETs and prognosis of patients with solid cancers. Pooled HR for OS and DFS demonstrated that high NETs levels were significantly associated with poor OS (HR: 1.80, 95% CI: 1.35–2.41, *P* < 0.0001) (11, 20, 25–28, 30, 32) and DFS (HR: 2.26, 95% CI: 1.82–2.82, *P* < 0.00001) (**Figure 2**).

Subgroup analysis based on sample type demonstrated that the association between high NETs levels and poor prognosis was consistent, irrespective of sample type (**Figure 2**). When subgroup analyses were conducted according to the primary organ site (**Supplementary Figure S2**), the included studies were classified into gastrointestinal cancers [stomach (29, 30), n = 3; rectum (11, 24, 31), n = 3], hepatobiliary cancers [pancreas (21, 22), n = 2; liver (20), n = 1], and other malignancies [breast (33), urinary bladder (25), and uterine cervix (23), n = 1 each]. Within these categories, elevated levels of NETs were consistently associated with poorer prognosis. In contrast, three studies (26, 27, 32) evaluated the prognostic role of NETs across a broad spectrum of malignancies. In this heterogeneous cohort, no significant association between NETs and prognosis was identified. Except for the study by Martinez–Cannon et al. (33), all the included studies conducted multivariate analyses and demonstrated a significant association between high NET levels and pooled HRs (**Figure 2**; **Supplementary Figure S3**). As Martinez–Cannon et al. (33) did not report HR, data from their Kaplan–Meier curves were extracted and analyzed using the method described by Parmar et al. (34). The analysis revealed no significant association between high NETs and prognosis (**Supplementary Figure S3**). Subgroup analyses stratified according to ethnicity consistently showed that high NET levels were associated with a poor prognosis across all ethnic groups (**Supplementary Figure S4**).

### Subgroup analyses by detection methods and antibodies used for NETs analysis

**Figure 3:**
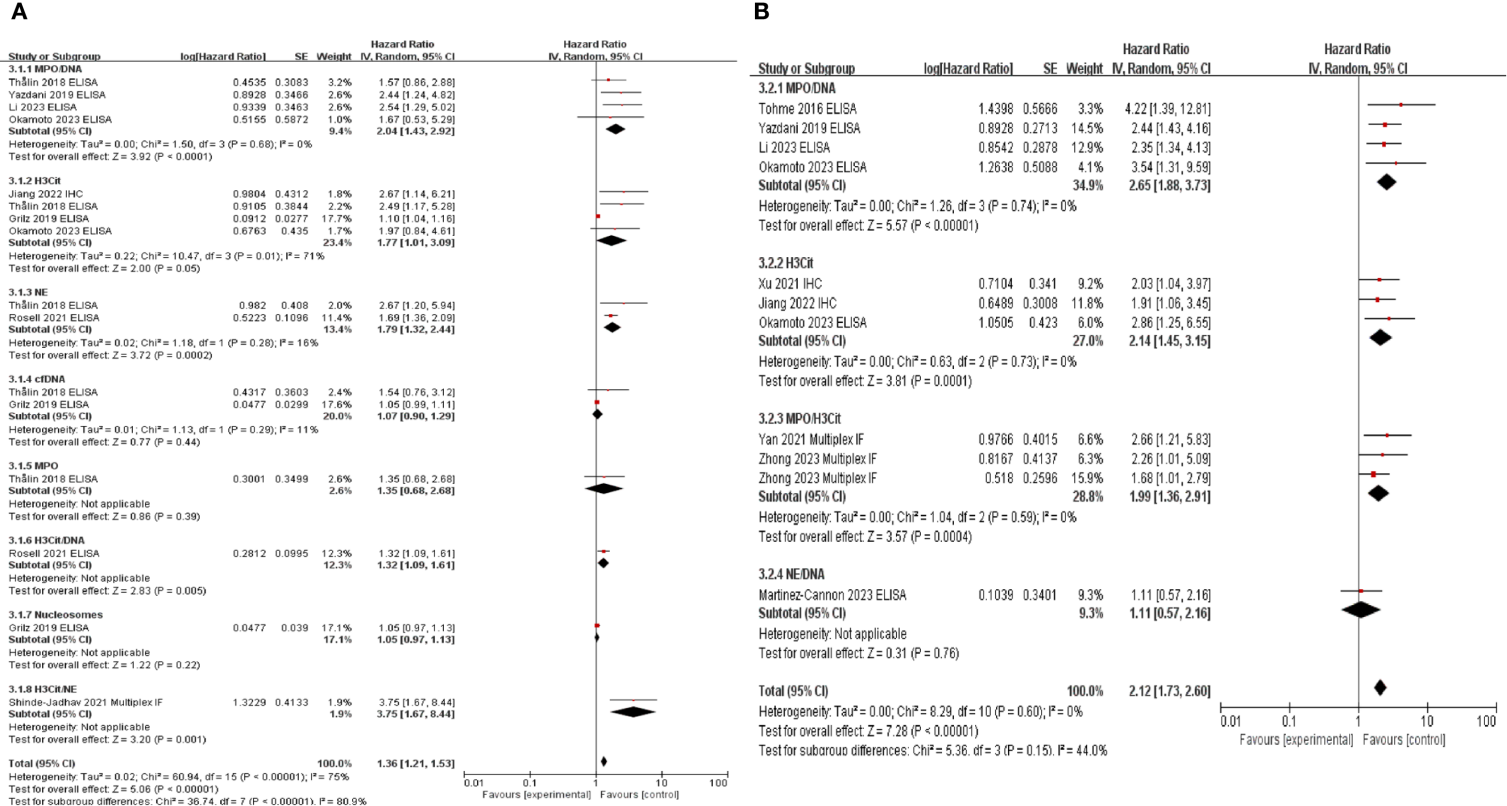
Subgroup analysis according to neutrophil extracellular traps detecting antibodies. Subgroup analysis was performed to evaluate overall survival **(A)** and disease-free survival **(B)** based on the antibodies used to detect neutrophil extracellular traps.

For tissue samples, IHC with H3Cit alone or multiplex IF with co-staining with MPO/H3Cit or NE/H3Cit was utilized. The most frequently used assay for blood samples was ELISA for the MPO/DNA complex, with H3Cit measurement being the next most common method. In the OS analysis, MPO/DNA (HR: 2.04, 95% CI: 1.43–2.92, *P* < 0.0001), H3Cit (HR: 2.37, 95% CI: 1.48–3.78, *P* = 0.0003), NE (HR: 1.79, 95% CI: 1.32–2.44, *P* = 0.0002), and H3Cit/NE co-staining (HR: 3.75, 95% CI: 1.67–8.44, *P* = 0.001) demonstrated significant associations, while for DFS, MPO/DNA (HR: 2.65, 95% CI: 1.88–3.73, *P* < 0.00001), H3Cit (HR: 2.14, 95% CI: 1.45–3.15, *P* = 0.0001), and H3Cit/MPO co-staining (HR: 1.99, 95% CI: 1.36–2.91, *P* = 0.0004) showed significant associations. Notably, MPO-DNA and H3Cit levels were associated with both OS and DFS, indicating their potential as key biomarkers. In contrast, NE-DNA (*P* = 0.97) and cfDNA (*P* = 0.44) levels were not significantly different. Furthermore, MPO, H3Cit-DNA, nucleosomes, and NE-DNA were evaluated in only one study, limiting the feasibility of further analyses (**Figure 3**).

### High NETs levels and clinicopathological parameters

**Figure 4:**
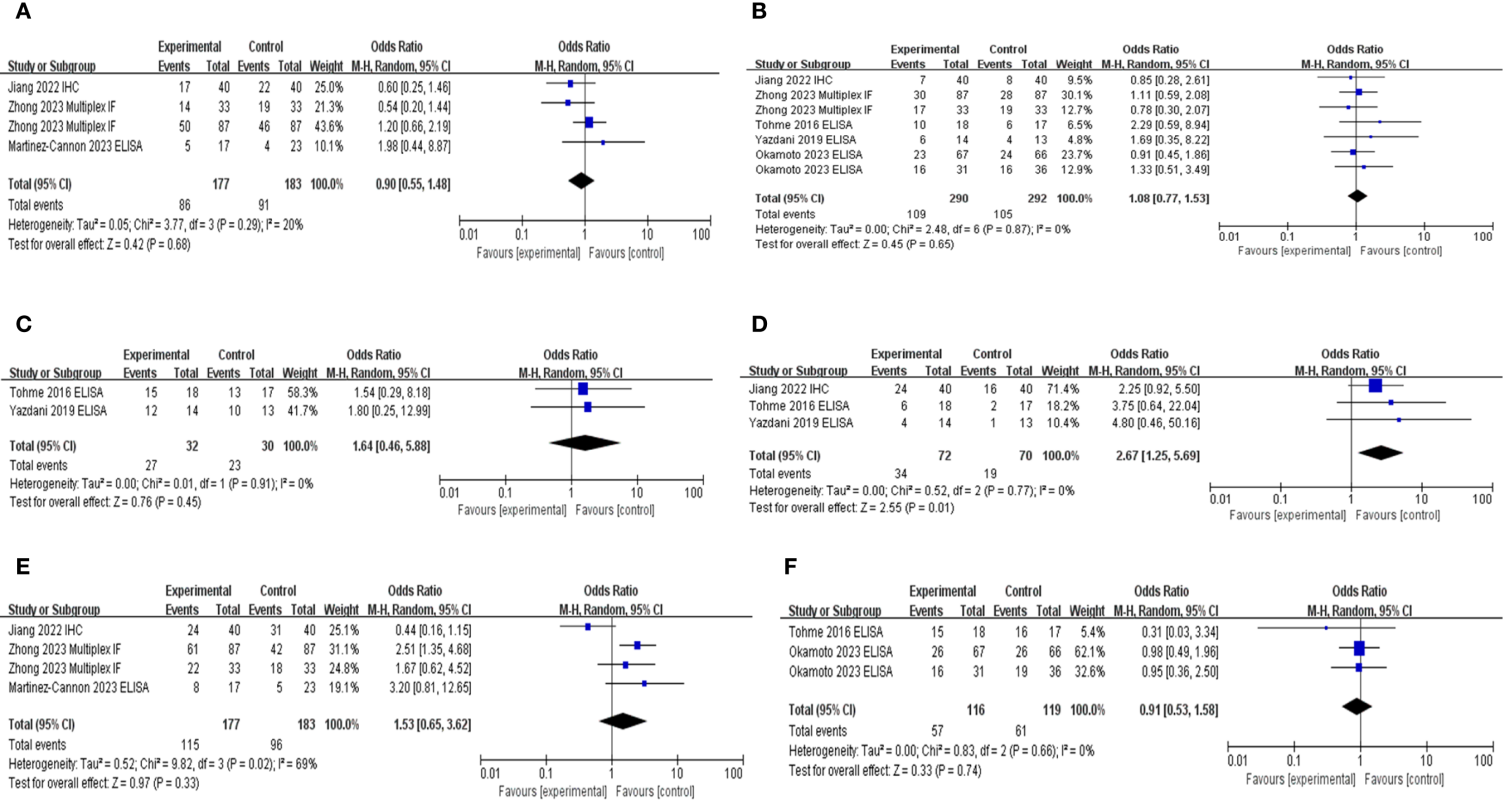
Subgroup hazard ratio analysis of neutrophil extracellular traps and pathological parameters in patients with cancer: **(A)** age, **(B)** sex, **(C)** American Society of Anesthesiologists classification, **(D)** tumor size, **(E)** TNM stage, and **(F)** adjuvant chemotherapy.

Principal clinicopathological parameters associated with elevated NETs levels, as reported across all studies included in the meta-analysis, are summarized in **Table 2**; **Supplementary Table S2**; **Figure 4**. The pooled analysis indicated that larger tumor size (OR: 2.17; 95% CI: 1.28–5.74; *P* = 0.02) and advanced TNM stage (OR: 1.63; 95% CI: 1.07–2.50; *P* = 0.0003) were significantly associated with high NETs levels (**Table 2**).

**Table 2:** Summary of the meta-analysis evaluating the relationship between neutrophil extracellular traps and clinicopathological parameters.

Parameters	Number of studies	Number of patients	Pooled OR (95% CI)	*p*-value	Heterogeneity		
					I^2^(%)	*P* -value	Model
Age (older)	3	360	0.92 [0.60, 1.40]	0.70	20%	0.29	Fixed
Sex (Female)	5	582	1.08 [0.77, 1.53]	0.65	0%	0.87	Fixed
TNM stage (advanced)	3	360	1.63 [1.07, 2.50]	0.02	69%	0.02	Fixed
ASA (3,4)	2	62	1.64 [0.46, 5.88]	0.45	0%	0.91	Fixed
Adjuvant chemotherapy (Yes)	2	235	0.96 [0.56, 1.65]	0.88	0%	0.63	Fixed
Tumor size (≥5 cm)	3	142	2.71 [1.28, 5.74]	0.009	0%	0.77	Fixed

OR, Odd ratio; CI, Confidence interval; ASA, American Society of Anesthesiologists classification.

## Discussion

Our analysis confirmed that elevated NETs levels were associated with poor prognosis in patients with cancer, regardless of whether tissue or blood samples were analyzed (**Figure 2**). This association remained consistent across various detection methods and most antibodies used, with the exception of cfDNA-based approaches, thereby underscoring the prognostic relevance of NETs in oncology (**Figure 3**; **Supplementary Figure S5**). To the best of our knowledge, this is the first comprehensive evaluation of the association between NETs and cancer outcomes, suggesting their potential clinical utility in cancer treatment and management.

The objective of this review was to comprehensively identify studies that have investigated NETs across all cancer types. The low inclusion rate (0.48%) following literature screening reflects the rigorous selection criteria applied to ensure the inclusion of studies specifically addressing the role of NETs in various malignancies. Despite extensive efforts, the limited number of studies reporting PFS (22, 29) and CSS (22) outcomes, likely reflecting the early stages of NET studies in oncology, has restricted our ability to conduct detailed subgroup analyses. Additionally, an insufficient number of studies investigating MPO (27), H3Cit/DNA (32), nucleosomes (26), NE/H3Cit (25), and NE/DNA (33) were available, precluding meaningful subgroup analyses for these markers (**Figure 3**; **Supplementary Figure S5**). Therefore, expanding the scope of future studies will be essential in providing a more comprehensive understanding of the role of NETs in cancer progression.

NETs are web-like structures composed of chromatin filaments coated with histones, proteases, and various granular and cytosolic proteins. NETosis is the process in which neutrophils generate and release NETs. This mechanism facilitates the immobilization and capture of pathogens, including bacteria, fungi, and viruses, thereby enhancing the efficiency of host antimicrobial defense (4–7). Recently, the role of NETs in various cancers has garnered increasing attention (6–8). Investigations of the antitumor functions of NETs have been conducted in colorectal cancer (36), head and neck squamous cell carcinoma (37), and malignant melanoma (38), predominantly using *in vitro* experimental studies. These studies suggested that NETs exert their antitumor effects by inducing apoptosis (36, 37) and necrosis (38).

In the present meta-analysis, we confirmed that elevated NET levels in both human tissues and blood samples were consistently associated with poor patient survival (**Figure 2**). This is likely due to the direct role of NETs in promoting tumor cell proliferation (11) and metastasis (12–14). Tumor-derived cytokines (e.g., interleukin [IL]-8, IL-17, granulocyte colony-stimulating factor, and CXCL6) recruit neutrophils and induce NETosis, thereby promoting tumor proliferation (39, 40). High mobility group box 1 (HMGB1), a NETs component, enhances proliferation by activating mitogen-activated protein kinase via toll-like receptor 9 (TLR9) and stimulating nuclear factor kappa B signaling (31) and IL-8 secretion through Receptor for Advanced Glycation End products (41). Additionally, NETs promote metastatic progression by degrading vascular endothelial-cadherin, thereby activating the Wnt/β-catenin signaling pathway and inducing the expression of epithelial–mesenchymal transition-related genes such as ZEB1 and Snail (42, 43). In parallel, HMGB1, a NET-associated component, facilitates tumor metastasis by activating TLR9, which in turn stimulates p38 and JNK signaling cascades, enhancing cancer cell migration and invasion (31).

Circulating NETs also enhance tumor cell survival by suppressing the cytotoxic activity of infiltrating CD8^+^ T (15). Additionally, NETs have emerged as key mediators of cancer-associated thrombosis, the second leading cause of death in patients with cancer having hypercoagulable states (16). Emerging evidence indicates that NETs promote cancer-associated thrombosis by enhancing the adhesion, activation, and aggregation of platelets and erythrocytes, leading to fibrin deposition and clot formation (17). This process is partially mediated by neutrophil-derived histones via TLR2- and TLR4-dependent platelet activation (18).

High-grade NETs have been associated with poor prognosis in studies that used various antibodies in tissue samples (20–25). Our analysis demonstrated that H3Cit (26–28), MPO-DNA (11, 27, 28, 30, 31), and NE (27, 32) were associated with poor prognosis, whereas cfDNA (26, 27) showed no such association. H3Cit, MPO-DNA, and NE are the key markers of NETs formation (8). H3Cit is produced by PAD4-mediated citrullination of histone H3, promoting chromatin decondenzation (44). MPO-DNA reflects NETs activity and contributes to metastasis and inflammation (45). NE released from neutrophil granules facilitate DNA decondenzation by cleaving histones (8, 44). However, cfDNA is a non-specific marker for NETs, as it detects extracellular DNA, regardless of origin (46). Although cfDNA can arise from NETosis (8), it is also released during apoptosis, necrosis, and erythroid precursor enucleation, and also from NET-like structures produced by eosinophils and macrophages (46).

This study has several limitations. First, non-English publications were excluded, potentially introducing a selection bias. Second, one study lacking HRs with 95% CIs required indirect data extraction, which may have affected accuracy. Third, limited data were available on the association between high NETs and CSS or PFS, warranting further investigation. Despite these limitations, our meta-analysis supports the prognostic significance of elevated NETs in patients with cancer.

Our analysis confirmed that elevated NETs levels were associated with poor prognosis in patients with cancer, irrespective of sample type (tissue or blood). This association was consistent across most detection methods and antibodies except for cfDNA-based approaches, highlighting the prognostic relevance of NETs in oncology. We believe that elevated NETs levels have potential as a prognostic biomarker and may contribute to risk stratification and personalized therapeutic approaches in precision oncology.

## Data Availability

The original contributions presented in the study are included in the article/**Supplementary Material**. Further inquiries can be directed to the corresponding author.
