# Supplementary material for: Neutrophil extracellular traps predict poor survival in cancer: a systematic review and meta-analysis of studies on tissue and circulating biomarkers
Source: Front Immunol. 2025 Oct 3;16:1676854. doi: 10.3389/fimmu.2025.1676854 (PMC12531144; doi:10.3389/fimmu.2025.1676854)
Supplement: Supplementary file 1 [file Table1.docx]

**Supplementary Tables**

**Neutrophil Extracellular Traps Predict Poor Survival in Cancer: A Systematic Review and Meta-Analysis of Studies on Tissue and Circulating Biomarkers**

**Supplementary Table S1**. Detailed overview of keywords used in the search strategy

| Data  base | Search | Keywords |
| --- | --- | --- |
| PubMed | #1 | "Neoplasms"[Mesh] |
|  | #2 | "Neoplasms"[TW] OR "Tumor"[TW] OR "Neoplasm"[TW] OR "Tumors"[TW] OR "Neoplasia"[TW] OR "Neoplasias"[TW] OR "Cancer"[TW] OR "Cancers"[TW] OR "Malignant Neoplasm"[TW] OR "Malignancy"[TW] OR "Malignancies"[TW] OR "Malignant Neoplasms"[TW] OR "Neoplasm, Malignant"[TW] OR "Neoplasms, Malignant"[TW] OR "Benign Neoplasms"[TW] OR "Benign Neoplasm"[TW] OR "Neoplasms, Benign"[TW] OR "Neoplasm, Benign"[TW] |
|  | #3 Combine | #1 OR #2 |
|  | #4 Combine | "Neutrophil Extracellular Traps"[TW] OR "Neutrophil Extracellular Trap"[TW] OR "NETs (Neutrophil Extracellular Traps)"[TW] OR "NET (Neutrophil Extracellular Traps)"[TW] OR "Netosis"[TW] OR "neutrophil extracellular trap"[TW] OR "neutrophil extracelluar traps"[TW] OR "neutrophils extracellular trap"[TW] |
|  | #5 Combine | #3 AND #4 |
|  |  |  |
|  |  |  |
| Data  base | Search | Keywords |
| EMBASE | #1 | "neoplasm"/exp |
|  | #2 | "Neoplasms":ti,ab,kw,de OR "Tumor":ti,ab,kw,de OR "Neoplasm":ti,ab,kw,de OR "Tumors":ti,ab,kw,de OR "Neoplasia":ti,ab,kw,de OR "Neoplasias":ti,ab,kw,de OR "Cancer":ti,ab,kw,de OR "Cancers":ti,ab,kw,de OR "Malignant Neoplasm":ti,ab,kw,de OR "Malignancy":ti,ab,kw,de OR "Malignancies":ti,ab,kw,de OR "Malignant Neoplasms":ti,ab,kw,de OR "Neoplasm, Malignant":ti,ab,kw,de OR "Neoplasms, Malignant":ti,ab,kw,de OR "Benign Neoplasms":ti,ab,kw,de OR "Benign Neoplasm":ti,ab,kw,de OR "Neoplasms, Benign":ti,ab,kw,de OR "Neoplasm, Benign":ti,ab,kw,de |
|  | #3 Combine | #1 OR #2 |
|  | #4 Combine | "Neutrophil Extracellular Traps":ti,ab,kw,de OR "Neutrophil Extracellular Trap":ti,ab,kw,de OR "NETs (Neutrophil Extracellular Traps)":ti,ab,kw,de OR "NET (Neutrophil Extracellular Traps)":ti,ab,kw,de OR "Netosis":ti,ab,kw,de OR "neutrophil extracellular trap":ti,ab,kw,de OR "neutrophil extracelluar traps":ti,ab,kw,de OR "neutrophils extracellular trap":ti,ab,kw,de |
|  | #5 Combine | #3 AND #4 |
|  |  |  |
|  |  |  |
| Data  base | Search | Keywords |
| Cochrane Library | #1 | [mh "Neoplasms"] |
|  | #2 | "Neoplasms":ti,ab,kw OR "Tumor":ti,ab,kw OR "Neoplasm":ti,ab,kw OR "Tumors":ti,ab,kw OR "Neoplasia":ti,ab,kw OR "Neoplasias":ti,ab,kw OR "Cancer":ti,ab,kw OR "Cancers":ti,ab,kw OR "Malignant Neoplasm":ti,ab,kw OR "Malignancy":ti,ab,kw OR "Malignancies":ti,ab,kw OR "Malignant Neoplasms":ti,ab,kw OR "Neoplasm, Malignant":ti,ab,kw OR "Neoplasms, Malignant":ti,ab,kw OR "Benign Neoplasms":ti,ab,kw OR "Benign Neoplasm":ti,ab,kw OR "Neoplasms, Benign":ti,ab,kw OR "Neoplasm, Benign":ti,ab,kw |
|  | #3 Combine | #1 OR #2 |
|  | #4 Combine | "Neutrophil Extracellular Traps":ti,ab,kw OR "Neutrophil Extracellular Trap":ti,ab,kw OR "NETs (Neutrophil Extracellular Traps)":ti,ab,kw OR "NET (Neutrophil Extracellular Traps)":ti,ab,kw OR "Netosis":ti,ab,kw OR "neutrophil extracellular trap":ti,ab,kw OR "neutrophil extracelluar traps":ti,ab,kw OR "neutrophils extracellular trap":ti,ab,kw |
|  | #5 Combine | #3 AND #4 |

| Table S2. Main characteristics of all studies included studies | | | | | | | |
| --- | --- | --- | --- | --- | --- | --- | --- |
| Sample Type | Authors Year | Country | Sampling year | Age  (Mean) | Pathological  Stage | Follow up  (Month) | Treatment |
| Tissue | Shinde-Jadhav et al., 2021 | Canada | 2004–2018 | 75 | T2, T3 | - | Radical surgery and radiation therapy |
|  | Xu et al., 2021 | China | 2013–2019 | 51 | I, II, III | 50.6 | Radical surgery |
|  | Yan et al., 2021 | China | 2010–2011 | 48 | I, II, III, IV | 64 | Radical surgery, chemotherapy, and radiotherapy |
|  | Chen et al., 2022 | China | 2015–2019 | - | I, II, III, IV | - | Radical surgery and adjuvant chemotherapy |
|  | Jiang et al., 2022 | China | 2010–2020 | 50 | I, II, III | - | - |
|  | Zhong et al., 2023 | China | 2015–2020 | - | I, II | - | Radical surgery, neoadjuvant chemoradiotherapy, and adjuvant chemotherapy |
| Blood | Tohme et al., 2016 | US | 2010–2012 | 62 | - | 24.3 | Radical surgery |
|  | Thålin et al., 2018 | Sweden | 2015–2017 | 70.4 | - | - | - |
|  | Grilz et al., 2019 | Austria | 2007–2013 | 61 | - | 24 | - |
|  | Yazdani et al., 2019 | US | 2010–2016 | 62.2 | I, II, III, IV | - | Radical surgery |
|  | Zhang et al., 2020 | China | 2019–2020 | - | I, II, III, IV | 5.5 | Radical surgery and adjuvant chemotherapy |
|  | Rosell et al., 2021 | Sweden | 2016–2018 | 73 | - | 6 | Palliative treatment |
|  | Li et al., 2023 | China | 2017–2019 | - | - | 12 | Radical surgery |
|  | Martinez–Cannon et al., 2023 | Mexico | 2017–2019 | 56.5 | I, IIA, IIB, IIIC | 43.5 | ^-^ |
|  | Okamoto et al., 2023 | Japan | 2006–2014 | 68.59 | I, II, III | - | Radical surgery |
| * Adjuvant chemotherapy; ^†^ low molecular weight heparins; ^‡^ Neutrophil Elastase Inhibitor | | | | | | | |

| Table S3. Characteristics of patient’s clinicopathological data included in the meta-analysis | | | | | | | | | | | | | | | | |
| --- | --- | --- | --- | --- | --- | --- | --- | --- | --- | --- | --- | --- | --- | --- | --- | --- |
| Author/Year | Tohme  2016 | | Yazdani  2019 | | Jiang  2022 | | Martiniz-cannon  2023 | | Okamoto  (Cit-H3)  2023 | | Okamoto  (MPO-DNA)  2023 | | Zhong  (Training group)  2023 | | Zhong  (Validation group)  2023 | |
| NETs  (low vs. high) | 17 | 18 | 13 | 14 | 40 | 40 | 23 | 17 | 66 | 67 | 36 | 31 | 87 | 87 | 33 | 33 |
| Age  (older) | - | - | - | - | 18  (45%) | 23  (57.5%) | 4  (44.4%) | 5  (55.6%) | - | - | - | - | 46  (52.9%) | 50  (57.5%) | 19  (57.6%) | 14  (42.4%) |
| Sex  (Female) | 11  (64.7%) | 8  (44.4%) | 9  (69.2%) | 8  (57.1%) | 32  (80%) | 33  (82.5%) | - | - | 24  (36.4%) | 23  (34.3%) | 16  (44.4%) | 16  (51.6%) | 28  (32.2%) | 30  (34.5%) | 19  (57.6%) | 17  (51.5%) |
| ASA  (3,4) | 13  (76.5%) | 15  (83.4%) | 10  (76.9%) | 12  (85.7) | - | - | - | - | - | - | - | - | - | - | - | - |
| Tumor size  (≥5 cm) | 2  (11.8%) | 6  (33.3%) | 1  (7.7%) | 4  (28.6%) | 16  (40%) | 24  (60%) | - | - | - | - | - | - | - | - | - | - |
| TNM stage  (advanced) | - | - | - | - | 9  (22.5%) | 16  (40%) | 5  (38.5%) | 8  (61.5%) | - | - | - | - | 42  (48.3%) | 61  (70.1%) | 18  (54.5%) | 22  (66.7%) |
| Adjuvant chemotherapy  (Yes) | 16  (94.1%) | 15  (83.3%) | - | - | - | - | - | - | 40  (60.6%) | 41  (61.2%) | 17  (47.2%) | 15  (48.4%) | - | - | - | - |

NETs, neutrophil extracellular traps; ASA, American Society of Anesthesiologists classification; H3Cit, citrullinated histone H3; MPO, myeloperoxidase
