# Supplementary figures and images for: Neutrophil extracellular traps predict poor survival in cancer: a systematic review and meta-analysis of studies on tissue and circulating biomarkers
Source: Front Immunol. 2025 Oct 3;16:1676854. doi: 10.3389/fimmu.2025.1676854 (PMC12531144; doi:10.3389/fimmu.2025.1676854)

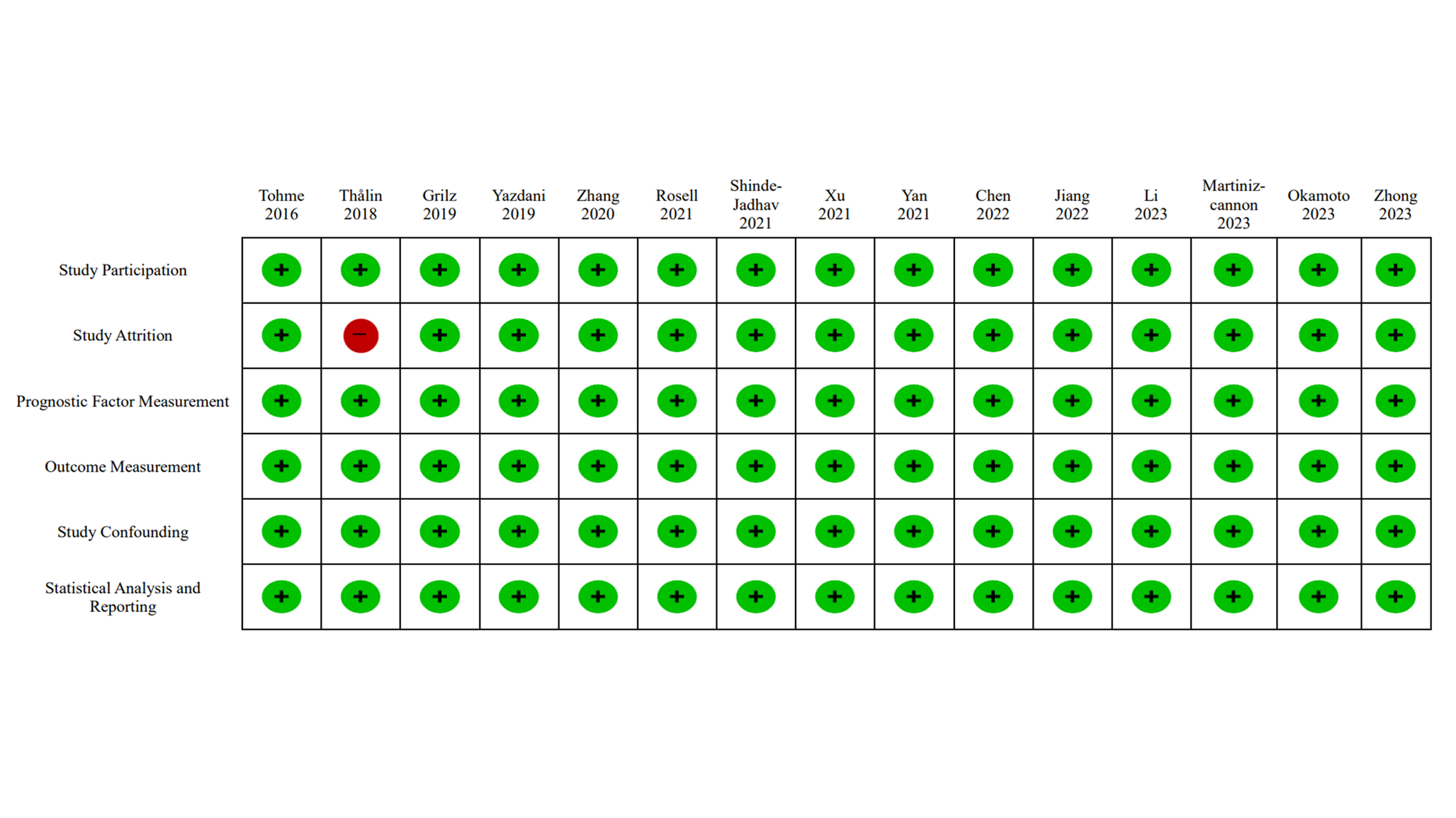

Supplement: Supplementary file 2 [file Image1.tiff]

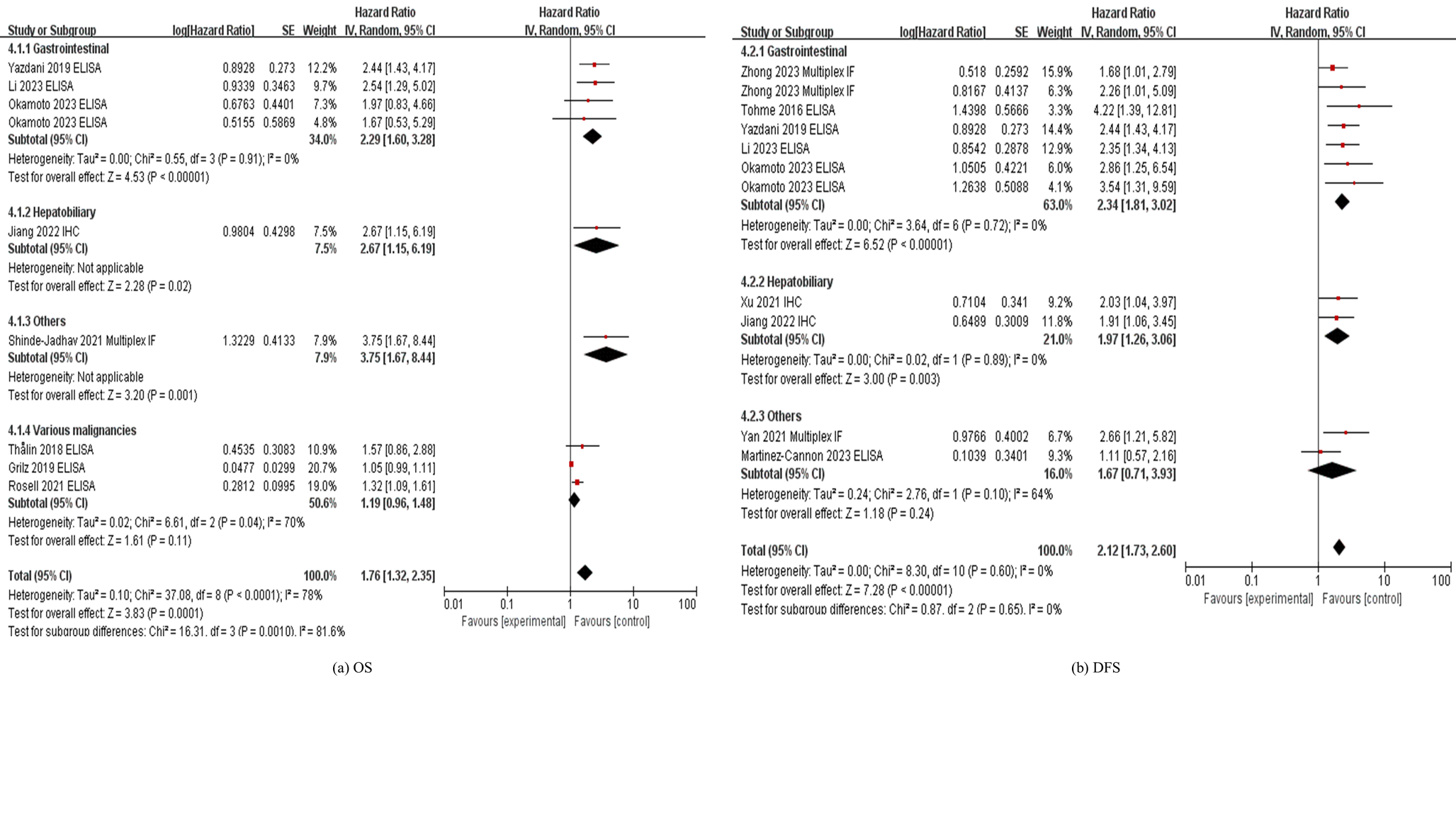

Supplement: Supplementary file 3 [file Image2.tiff]

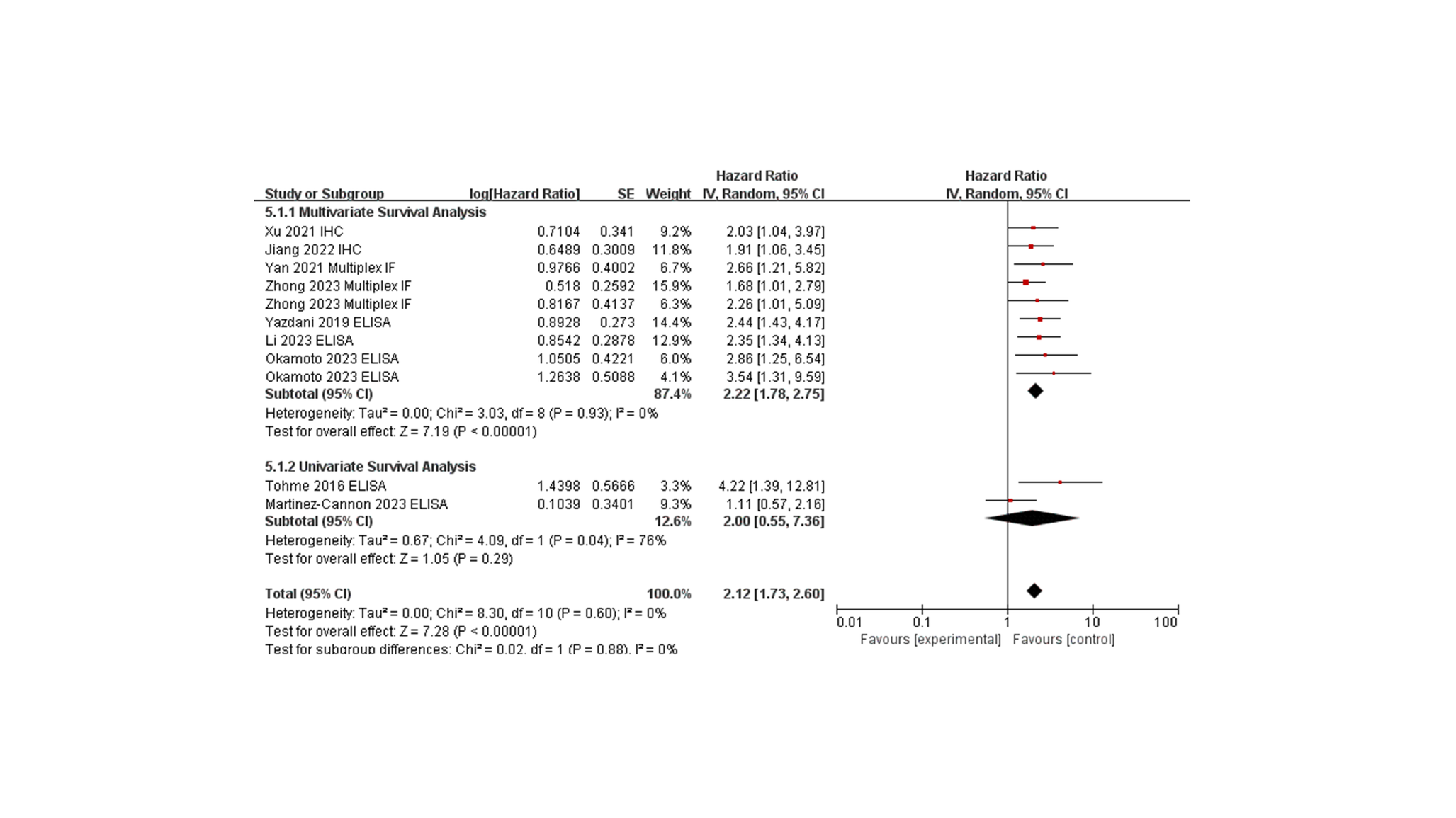

Supplement: Supplementary file 4 [file Image3.tiff]

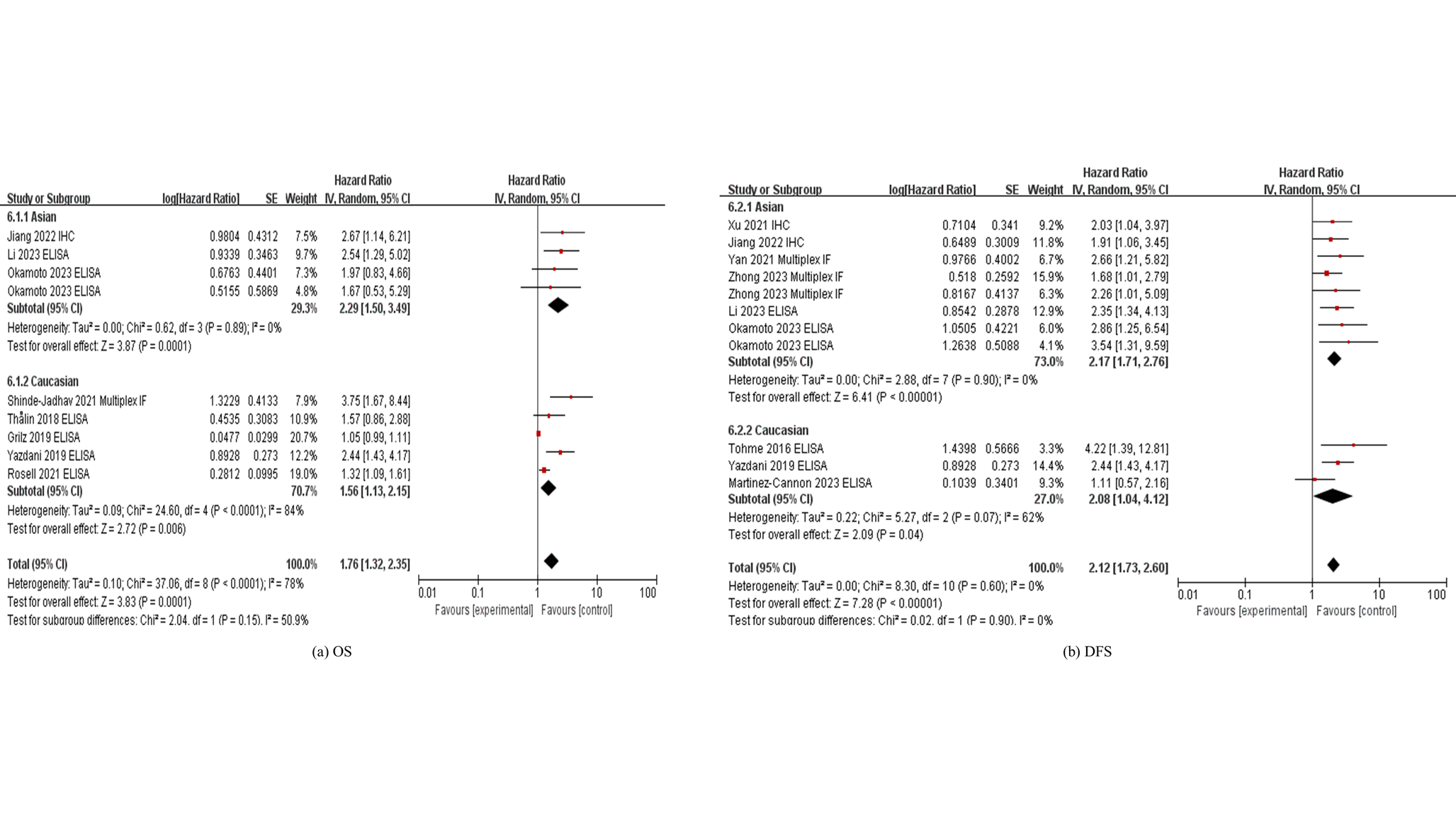

Supplement: Supplementary file 5 [file Image4.tiff]

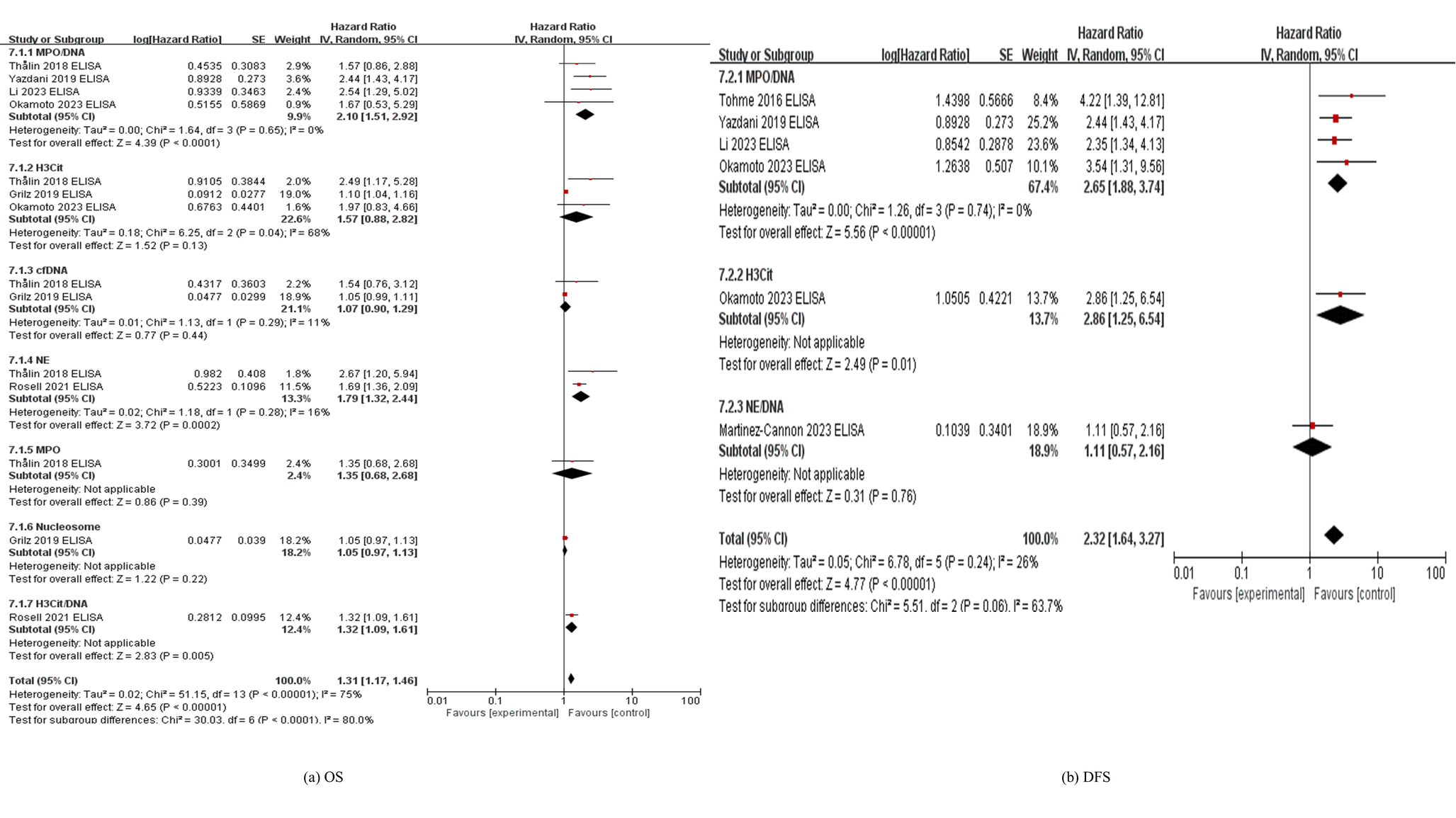

Supplement: Supplementary file 6 [file Image5.tiff]
